# Fluctuations in measured radioactive decay rates inside a modified Faraday cage: Correlations with space weather

**DOI:** 10.1038/s41598-020-64497-0

**Published:** 2020-05-22

**Authors:** V. Milián-Sánchez, F. Scholkmann, P. Fernández de Córdoba, A. Mocholí-Salcedo, F. Mocholí, M. E. Iglesias-Martínez, J. C. Castro-Palacio, V. A. Kolombet, V. A. Panchelyuga, G. Verdú

**Affiliations:** 1 Institute for Industrial, Radiophysical and Environmental Safety, Universitat Politècnica de València, Camino de Vera, s/n, 46022 València, Spain; 2 Research Office for Complex Physical and Biological Systems, 8006 Zurich, Switzerland; 3 Grupo de Modelización Interdisciplinar, InterTech, Instituto Universitario de Matemática Pura y Aplicada, Universitat Politècnica de València, Camino de Vera, s/n, 46022 València, Spain; 4 Traffic Control Systems Group, ITACA Institute, Universitat Politècnica de València, Camino de Vera, s/n, 46022 València, Spain; 5 Institute of Theoretical and Experimental Biophysics, Russian Academy of Science, Moscow Region, Pushchino 142290 Russia; 6 Chemical and Nuclear Engineering Department, Universitat Politécnica de València, Camino de Vera, s/n, 46022 València, Spain

**Keywords:** Experimental nuclear physics, Nuclear astrophysics, Magnetospheric physics

## Abstract

For several years, reports have been published about fluctuations in measured radioactive decay time-series and in some instances linked to astrophysical as well as classical environmental influences. Anomalous behaviors of radioactive decay measurement and measurement of capacitance inside and outside a modified Faraday cage were documented by our group in previous work. In the present report, we present an in-depth analysis of our measurement with regard to possible correlations with space weather, i.e. the geomagnetic activity (GMA) and cosmic-ray activity (CRA). Our analysis revealed that the decay and capacitance time-series are statistically significantly correlated with GMA and CRA when specific conditions are met. The conditions are explained in detail and an outlook is given on how to further investigate this important finding. Our discovery is relevant for all researchers investigating radioactive decay measurements since they point out that the space weather condition during the measurement is relevant for partially explaining the observed variability.

## Introduction

In a previously published paper by our group we reported about the observation of anomalies in measured radioactive decay rates and capacitance measurements inside a modified Faraday cage (MFC)^1^. In that paper we described the anomalous results obtained when measuring background radiation, radioactive decay rates of a set of different nuclides (beta emitters) with a Geiger-Müller (GM) counter tube, and of the gamma spectrum of a source with a multichannel analyzer (MCA). Statistically significant variations were found of the mean (or median) values and of other statistics as compared with the initial measurements taken outside the cage. These findings raised several questions about possible effects on the circuitry of the detectors, which in turn could lead to anomalous pulses shapes and hence to the observed anomalous measurements. To address these questions, and since the shapes of the pulses depend on the resistors (R) and capacitors (C) used in the sensors^2^, we also investigated the response of a low-pass RC filter, the stability of a class I multilayer ceramic capacitor, and the stability of the connecting cables capacitance when placed inside the box. We found significant changes in the low-pass filter time constant and in the stability of the capacitances’ values.

Possible influences of environmental parameters like ambient temperature, pressure and humidity on the detectors’ stability were considered in a next step. Similarly, as in a series of experiments reported in the literature (performed in the field of decay rates variability)^3,4^, environmental factors were analyzed but no obvious correlations could be detected. We concluded that the variability in the measurements could not be fully explained by variations in those environmental parameters, and if those parameters would have remained stable, the referred anomalies would have been even greater^1^. Consequently, none of the up to now referred factors could explain all the observed variability in the measurements.

Some experiments and studies about measured radioactive decay rates variability suggest that non-classical environmental factors, mostly cosmophysical ones, correlate with the variability. With this respect, studies were published linking nuclear decay variations with solar activity via solar neutrinos^5^, and/or with cosmic (relic) neutrinos of very low energy^6,7^. In addition, Pons and Baurov proposed further astrophysical factors influencing the decay^8–14^. These findings and conclusion are controversially discussed and experimental results were published that seemingly contradict them^15–26^, but the controversy persists^27–34^. According to Elmaghraby^35^, both viewpoints could be reconciled because the different results can be attributed to the differences in measuring systems, which can be or cannot be able to detect the energy involved in the interaction between neutrinos and nuclei.

On the other hand, the intensive research over many decades by Shnoll *et al*. suggests that there exist some unknown cosmophysical factor which affects the decay processes^36,37^. One example of this can be seen in the periodicity of the fine structure of the histograms of noise processes^36,38,39^. This means that when comparing the successive histograms one can observe that the similarity of the histograms shapes tends to be reproduced in time intervals, which are consecutive to each other (this is the “near-zone-effect” (NZE)). In this regard, it is worth noting that this effect can be correlated with some solar-geophysical indices^40^. Based on these correlations and on the observations by Jenkins and Fischbach^41^, we already performed a preliminary analysis looking for correlations with our data with cosmological factors and found some (i.e. peaks in decay rates close or coincidental with solar noon, solar midnight and with a solar storm)^1^.

A closer analysis of the correlation of our measured data with cosmophysical factors were performed by one of the authors (F.S.) and two initial report were publish by our group about this investigations^42,43^. It was found that the measured decay and capacitance data were, in some instances, significantly correlated with geomagnetic activity (Dcx index^44,45^), and/or with the activity of cosmic-ray induced neutrons^46–48^. In the present paper we show the rest of the measured results presented in our initial publication^1^ along with the space weather data.

In this paper, rather than attempting to present an explanatory conjecture of these results (which will be tried in a next work), we first only present an as much as possible detailed description of the circumstances or conditions under which those correlations occurred or not occurred.

This paper aims to be a step forward in determining the physical factors that cause or trigger the observed anomalous effects inside the MFC. Therefore, more correlations with other indices should be investigated in future works, in a similar way to what was performed by Zenchenko *et al*.^40^ and^49^. This might lead to the characterization and modeling of such anomalous effects.

## Materials and Methods

In our previous publication^1^ a detailed description of the experiment, the setup and materials used was given. Briefly, four radioactive sources of ^226^Ra, ^204^Tl, ^90^Sr/^90^Y and ^137^Cs were used. These isotopes were chosen due to their easy availability and safe handling, as well as to have isotopes with both decay modes (alpha + alpha/beta chain: ^226^Ra, beta: ^204^Tl, ^90^Sr/^90^Y and ^137^Cs).

In this paper we will refer to the results obtained with the three first sources which were used with a Geiger-Müller (GM) counter. The sources were always placed in the same position, as was the enclosure and the GM counter in the inside.

Based on the results explained in our previous publication^42^, the Dcx index was chosen in this study as a parameter which quantifies the state of the geomagnetic activity (GMA). The Dcx index can be low (i.e. Dcx = [−50 nT, 0 nT]), modest (i.e. Dcx = [−100 nT, −50 nT]) or strong (i.e. Dcx = [−250 nT, −100 nT]). In addition, we used the cosmic-ray induced neutron counts data (CRN) to capture the cosmic ray activity (CRA). Hourly data of the Dcx index and of neutron counts were obtained from http://dcx.oulu.fi and from http://www.nmdb.eu/nest/search.php, respectively.

The correlations between the measured data and the GMA and CRA indices were quantified by the Pearson correlation coefficient. Statistical significance testing was performed using Bayesian statistical analysis (JASP, version 0.9.2) resulting in Bayes factors (BF), indicating the evidence level for the alternative hypothesis (H_1_) and the null hypothesis (H_0_). The interpretation of the BF is as follows: >100: extreme evidence for H_1_, 30–100: very strong evidence for H_1_, 10–30: strong evidence for H_1_, 3–10: moderate evidence for H_1_, 1–3: anecdotal evidence for H_1_, 1: no evidence for H_1_ as well as H_0_, 1/3-1: anecdotal evidence for H_0_, 1/3-1/10: moderate evidence for H_0_, 1/10-1/30: strong evidence for H_0_, 1/30-1/100: very strong evidence for H_0_, <1/100: extreme evidence for H_0_.

Regarding the statistical test, there is a conceptual problem with the statistical testing of time-series correlations: it works only if there are not strong transients in the data. Since some of our data have such transients (for example, see Fig. 6a,b) the test indicates no correlation but in fact there is a clearly visible correlation. A statistically significant correlation between two time-series is therefore a sufficient but not necessary condition for the existence of a real correlation when the data are complex and nonlinear.

## Results and discussion

In this section, data of decay rates (or background counts) and capacitance measurements taken inside the MFC, which were presented in^1^, are compared with indices of GMA and CRA. These indices are two different aspects of space weather, both affected by various solar and interplanetary parameters^50–55^.

In the following figures, those decay (and capacitance) data are plotted against GMA and CRA variability. After the presentation of the figures of^1^ which were possible to analyze, the results of the correlation analyses are given. Later on, a description of the observed circumstances under which the correlations take place is also given. This refers to a description of the radioactive source used (whenever necessary), and of the state (and evolution) of the registered decay (or background) counts (in cpm), or the capacitance values. It may comprise a description of the state of the analyzed values relative to their initial values (outside the box, or just after its introduction in the inside), or of their subsequent variations and tendency along the analyzed period, i.e., how the decay rates or the capacitance evolve. As presented in^1^, those values can be higher, lower, or be the same compared to the initial values outside the cage (it should to be stressed that this aspect will be checked again in the next experiments, but in any case, the relevant fact is that the measures showed significant variations during the observation periods). Besides, they can have an increasing or decreasing trend, and/or show oscillating values, some of which may in turn differ significantly from each other.

Here, we consider that the system consists of the connected GM counter (or the capacitance) along with the radioactive preparation and the box: this is so because, as we could see in^1^, the presence of the box along with its structure influences the sensors’ responses (i.e., the GM counter and the capacitance measurements).

Subsequently, the description of the sensors’ response is accompanied by a description of how GMA and CRA vary, and on whether the sensors’ response coincides or not with the existence of a correlation with these factors. Thus, this set of factors could be considered as providing a phenomenological picture of the circumstances (situations/”states”) observed until now in which the correlations can take place or not (this picture is provisional due to the very limited amount of data). Besides, this is not an explanation of why the observed effects took place but could constitute a first step to achieve that goal.

### Background radioactivity and decay rates variations *vs*. geomagnetic and cosmic ray activity

#### Background radioactivity vs. geomagnetic and cosmic ray activity

**Figure 1:**
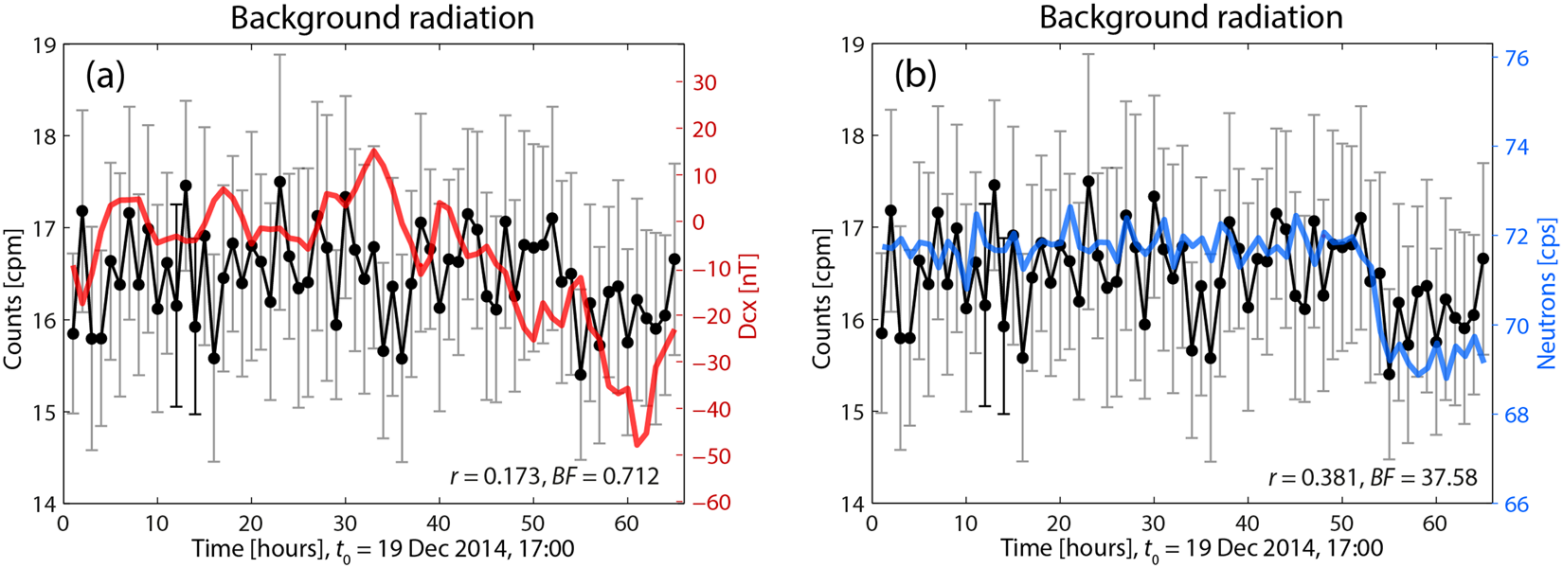
Background radiation *vs* GMA and CRA. Bck values (black) are increased compared to the first measurements outside the box. The indices seem to be not correlated. (**a**) There is no correlation between Bck and Dcx (red), although by visual inspection one can consider that there is a correlation. Most Dcx data oscillate close to 0 nT, and some other in the region between 10 nT and −50 nT; these data do not reflect any significant storm during the measuring period. (**b**) Neutron counts oscillate smoothly but close to 72 cps and are correlated with Bck.

The first comparisons with the cosmophysical factors referred to the background registered by the counter, which in turn was placed inside the box. Figure 1(a) shows the variability of background radioactivity (Bck in cpm) *versus* GMA (Dcx index, in nT), and in Fig. 1(b)*versus* CRA (neutron counts, in cps; see description about Fig. 1(d) in^1^). Background measurements were taken with the counter tube GM1 and there were no radioactive sources inside the MFC. The statistical analysis yields that there is no correlation between Bck and GMA (*r* = 0.173, BF = 0.712), but between Bck and CRA (*r* = 0.381, BF = 37.58). However, a similarity of the trends of Bck and GMA can be seen visually, as if they were slightly correlated. It is remarkable that the decrease in background counts in the last part of the time-series agrees with a decrease happening also in the GMA and CRA. The decrease is in-phase, as visually most evident in Fig. 1(b).

In respect of the observed background counts, using a GM counter, the experiment started by counting the background at the outside of the box, which yielded the initial values (cpm_0_ ~ 15.5 cpm). After placing the GM counter inside the box, counts increased to values above 17 cpm and behaved as shown in the figure. The characteristic of the decay counts being increased (relatively to their values outside the box) seems to be an important condition to observe some correlation. Besides, these background data are *oscillating* in such a way that some values are statistically different from others (skewness of this data set is 7.3). At the end of the time-series shown in Fig. 1, those values have a slightly decreased level, but are still high, thus showing a certain stationarity.

Regarding the GMA, the observed additional aspect is that its values are most of the time slightly around 0 nT or between 0 nT and −50 nT. This values distribution is apparently another factor that should be taken into account: under these circumstances, although the background cpm are anomalously increased (relatively to cpm_0_), there is, in principle, no correlation with Dcx; the lack of correlation has also to be contrasted with the state of CRA, because this factor could be dominant.

With regard to the correlation with CRA, its oscillations are low (the amplitudes of the jumps from one measurement to the next are usually of about 2 cpm), and run almost in parallel with the Bck, but its values are close or above 72 counts per second (which seems also to be another important reference value). Besides, this existing correlation with the background radiation counts *coincides with a state of increased number of neutron counts* and shows that when GM counts (in cpm) are increased and Dcx varies in a similar way as just seen (in a smooth way with most values close to 0 nT and above −50 nT), and without a correlation between Dcx and neutron counts, a correlation between the background data and CRA can take place That is to say, in this case, it could be thought that CRA dominates because the GMA is not “strong” enough (it remains close to 0 nT, where the index is low), whereas most of neutron count values are close or higher than 72 cpm.

All this will be seen again in the next cases. Besides, it will also be seen later that it can happen differently, i.e., GM counts can be correlated with Dcx and not with neutron counts (see Fig. 3), as if one of both indices were the dominating factor depending on the circumstances (in what follows other cases are described). However, hypothetically, this also could mean that a state of transition between the two situations could have been in progress, during which no correlation would be observed.

To recapitulate, the results presented in this section can be associated with the following:

(i) radiation counted (as background) is increased in more than 11% with respect to the initial counts in the outside;
(ii) Dcx varies mainly around 0 nT and in the range [10, −50] nT and is not correlated with the background data;
(iii) on the other hand, since background data and neutron counts are correlated and the neutron count values are very close to 72, one can conjecture that in this case CRA is strong enough to be the leading factor;
(iv) one should also notice that GMA and CRA are not correlated.

#### Radioactive decay of ^226^Ra vs. geomagnetic and cosmic-ray activity

**Figure 2:**
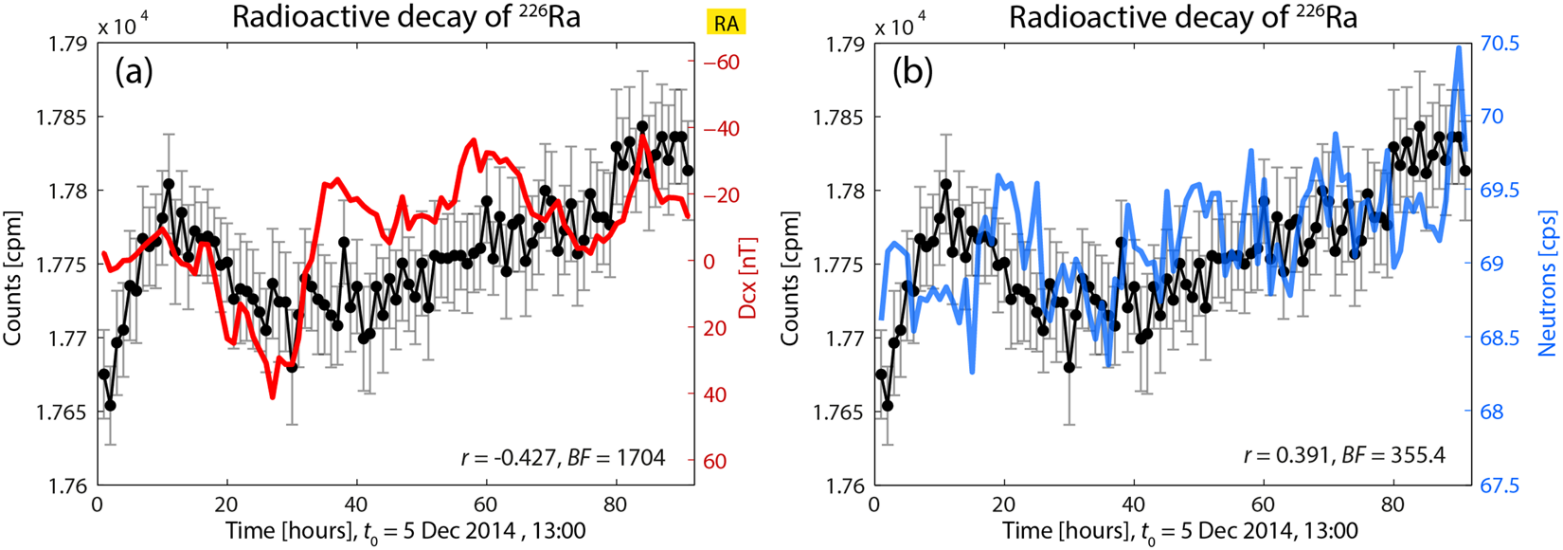
Decay rate of ^226^Ra (only this time obtained with GM4) *vs*. GMA and CRA. Decay data are increased above the values outside the MFC. Dcx and neutron counts are correlated, and there is a statistically significant correlation between decay rates and both indices. (**a**) Decay rates *vs*, Dcx. Dxc varies broadly between −40 and +40 nT. (**b**) The neutron count rate varies with greater amplitude than in Fig. 1. RA: reversed axis scaling.

**Figure 3:**
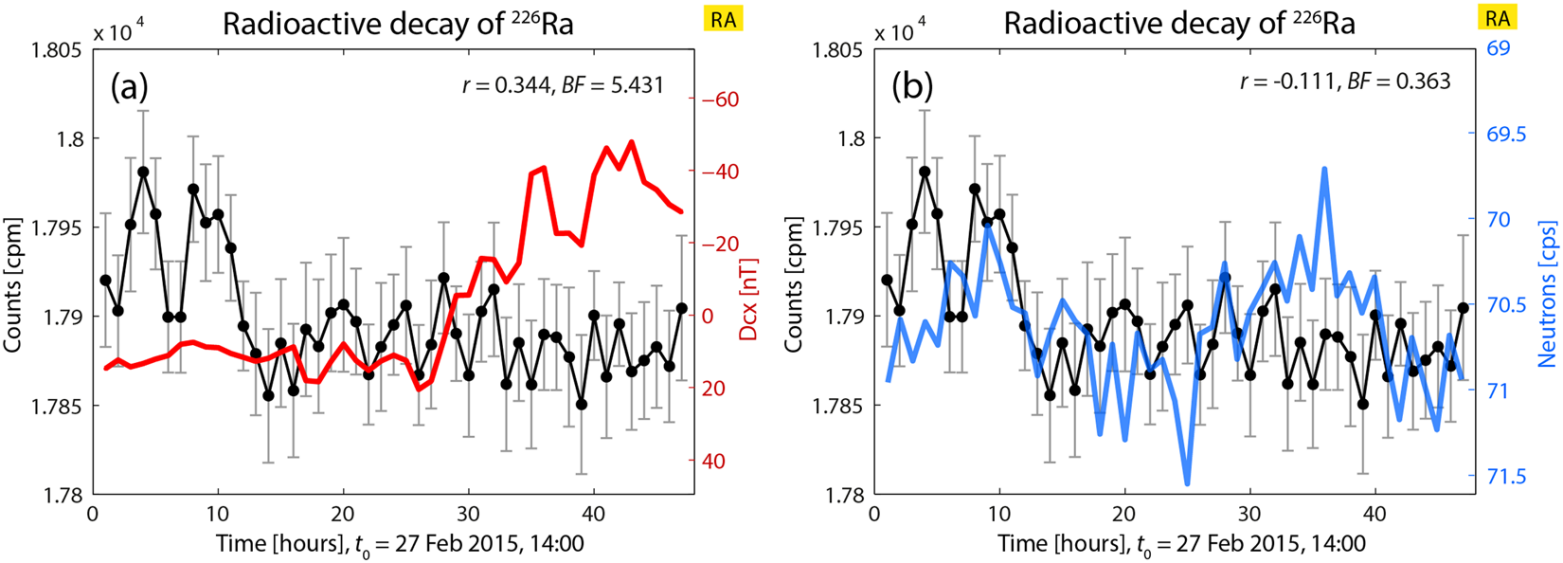
Decay rate of ^226^Ra (taken with GM1 inside the MFC) vs Dcx (**a**) and neutron counts (**b**). Decay rates inside the MFC are greater than the values outside the box. Correlation only exits between decay and Dcx.

Figure 2 shows the radioactive decay of ^226^Ra versus GMA and CRA (the decay was measured only in this experiment with counter tube GM4). The decay values of this figure were presented in Fig. 3a of our previous publication^1^. The correlation analysis resulted in the detection of a statistically significant correlation for both cases (decay *vs*. GMA: *r* = −0.427, BF = 1704; decay *vs*. CRA: *r* = 0.391, BF = 355.4).

In Fig. 2 one can observe that the measured decay values evolve from the initial 17675 cpm (as explained in^1^; this first initial value is the same as the values measured previously outside the box). The subsequent effect after placing the source inside the box consisted in a combination of (significantly) oscillating and increasing trend of the ^226^Ra decay data. These decay data were significantly higher (i.e. about 0.8%) than the initial ones and followed the variations experienced by Dcx and neutron counts. However, it should be noticed that the variables cpm and Dcx are anti-correlated.

It seems therefore that whatever the mechanism acting on this system could be, all happens as if this kind of “interface” (the box) were able to transmit the GMA and CRA conditions from the outside to the “sensors”, but in such a way that the decay measurements follow the same trend (keeping in mind the mentioned anti-correlation). So, it can be seen here (and in the following cases), that a good correlation with one GMA or CRA exists when the measured decay values are significantly higher than the ones obtained outside the box (in this case, cpm were in a “state” of increasing trend), or at least, when they show significantly high oscillations.

It might be worth noting that the decay rate values reached two maxima: the first located 0.8% above the starting values, and the second at 0.9%.

Regarding the correlation with GMA, Dcx values are oscillating between +40 and −40 nT, in a wider range than in the previous case (Fig. 1(a)) and are not restricted mainly to values close around or slightly below 0 nT. Such behavior seems strong enough to be another favorable factor for the correlation to occur.

Concerning the correlation with CRA, the correlation seems to occur due to the existing correlation between GMA and CRA (although Dcx is anti-correlated). One might ask what would have happened if Dcx had oscillated like in Fig. 1, and whether in such case the correlation had also existed only with CRA. In other words, one might ask whether there is a dominating factor to which the decay rate responds when one of both GMA and CRA do not behave in an appropriate manner. So, on the basis of what was seen in Fig. 1, (and on what can be seen in Fig. 3), it seems that Dcx has to oscillate in a stronger way (and show an increasing trend) to be the leading factor (see below oscillations of Dcx in Fig. 3, where there is no correlation between Dcx and neutron counts, and where neutron count oscillations are more noticeable than in Fig. 1, but lower than 72 cpm). In any case, under these circumstances a good correlation with CRA also exists.

Another question refers to the thickness of the shield: Does the lower thickness of GM4 allow a greater influence of indices variations on decay measurements via the MFC?

**Figure 4:**
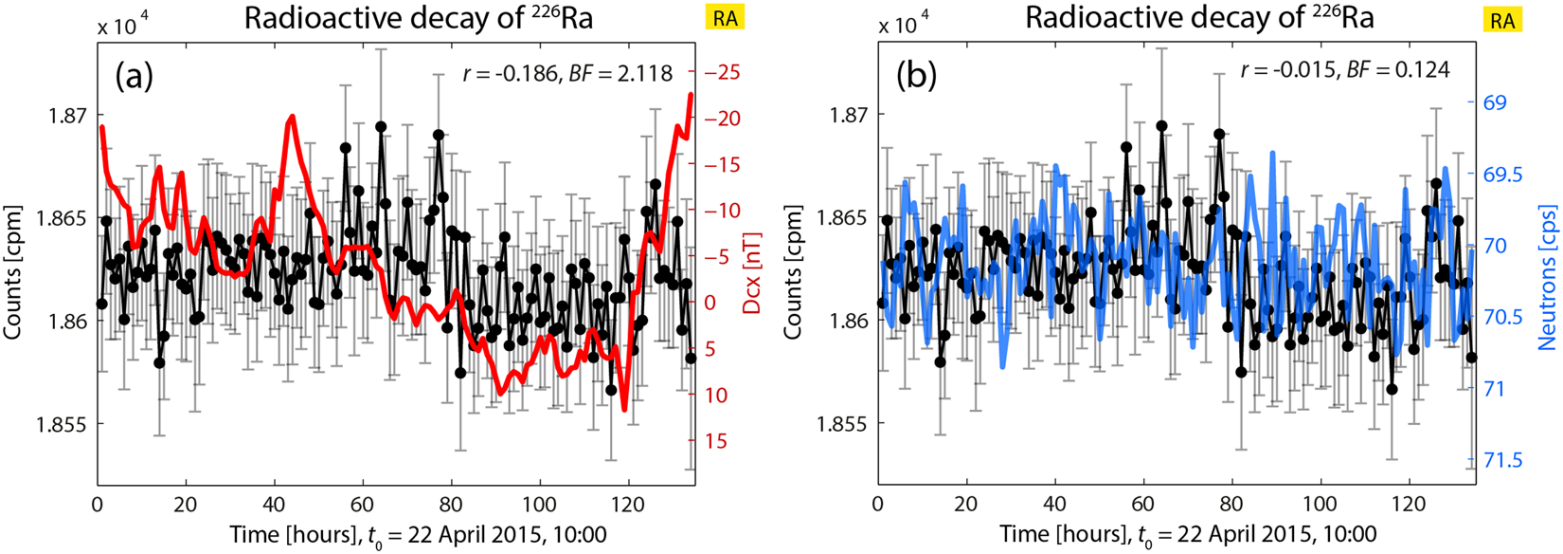
Correlations analysis between decay rates of ^226^Ra and Dcx (**a**) and neutron count data (**b**); the indices are not correlated. Decay data are increased with regard to the initial ones outside the box in about 4.3%. In (**a**), Dcx data oscillate less than in Fig. 2 but no correlation can be found. In (**b**) the oscillation pattern of neutron count data is similar to the one in Fig. 2, but there is no correlation with decay rate.

In Fig. 3 one can observe a case where a correlation only exists between decay rates of ^226^Ra (measured with GM1) and GMA (this figure is Fig. 4b in^1^) (decay vs. Dcx: *r* = 0.344, BF = 5.431; decay vs. neutron counts: *r* = −0.111, BF = 0.363). The Bayesian analysis yields that decay rate is statistically significantly correlated only with Dcx.

With regard to Fig. 3(a), it can be observed that the existing low correlation agrees with a combination of factors:

(i) decay counts at the beginning of the Fig. 3(a) increases only up to 0.4% with respect to the data at the beginning of this experiment (17900 cpm);
(ii) a large proportion of the decay data (about the last 75% of them) shows sustained and only slightly significant increased rates, and also shows some small oscillations with few values that are statistically different. Those data seem to have “returned” to the first values observed at the beginning of the experiments – as if the cage had lost “strength”, or “transmission capability” – (see first data in Fig. 2(a) in^1^). On the other hand, Dcx appears to oscillate in the range of [−50, 20] nT and to show variability (as in Fig. 2).

Regarding Fig. 3(b), the oscillations of the neutron counts are more intense than in Fig. 1. However, no correlation exists, coincidentally with values of the neutron counts which are lower than 71.5 and reduced to the range [70.5, 71.5], and in combination with the enclosure that does not yield increased decay rates, which again seems to be a determining factor.

In Fig. 4, depicting the results of another experiment, it can be appreciated a case where no correlation exists with none of the two indices, and where Dcx and neutron counts are not correlated. Here, the initial decay values are increased by about 4.3% relatively to the first measurements in 2014 June and July (these decay data were presented in Fig. 4(d) of^1^). Therefore, also in this case there is a “state” of initially very increased decay rates, along with a subsequent oscillating and slight decreasing trend.

In Fig. 4(a), the index Dcx oscillates showing noticeable variability and takes positive and negative values in the range [+10, −25] nT, with few values close to 0 nT; however no correlation with decay rates exist. Thus, in spite of the high increase in decay rates (which seemed to be a previous condition for the existence of a correlation between decay rates and Dcx when this index oscillates in this way), in this case no correlation appears.

In this case also, the state of increased decay rates does not seem to be an enough appropriate and strong previous condition (or the only condition) to find a correlation with GMA (decay vs. neutron counts: *r* = −0.186, BF = 2.118) or with CRA (decay *vs*. neutron counts: *r* = −0.015, BF = 0.124).

In this case, in spite of the variability of the neutron counts, which is stronger than in Fig. 1 but similar to that of Fig. 2(b), the variations of decay rate are not correlated with Dcx or neutron counts. This seems to be the opposite of what happens in Fig. 1 (where Dcx varies smoothly). From all the correlations discussed so far, we can conclude that when both indices were not correlated, the decay rate seemed to follow the most “dominant” factor, which up to now seemed to be Dcx when this index varies intensively enough. It might be conjectured that in this case both indices are strong enough to be the dominant factors (or the system might be in a state of transition), and therefore the decay counts are not correlated with either GMA or CRA.

On the other hand, Fig. 4 seems to show many strong transients in the plot of decay rate, an aspect that might prevent obtaining a correct evaluation of the correlation index.

Another conjecture about the lack of correlation is that although the decay counts are increased, Dcx and the neutron counts oscillate in a reduced range.

#### Radioactive decay of ^204^Tl *vs*. geomagnetic and cosmic-ray activity

**Figure 5:**
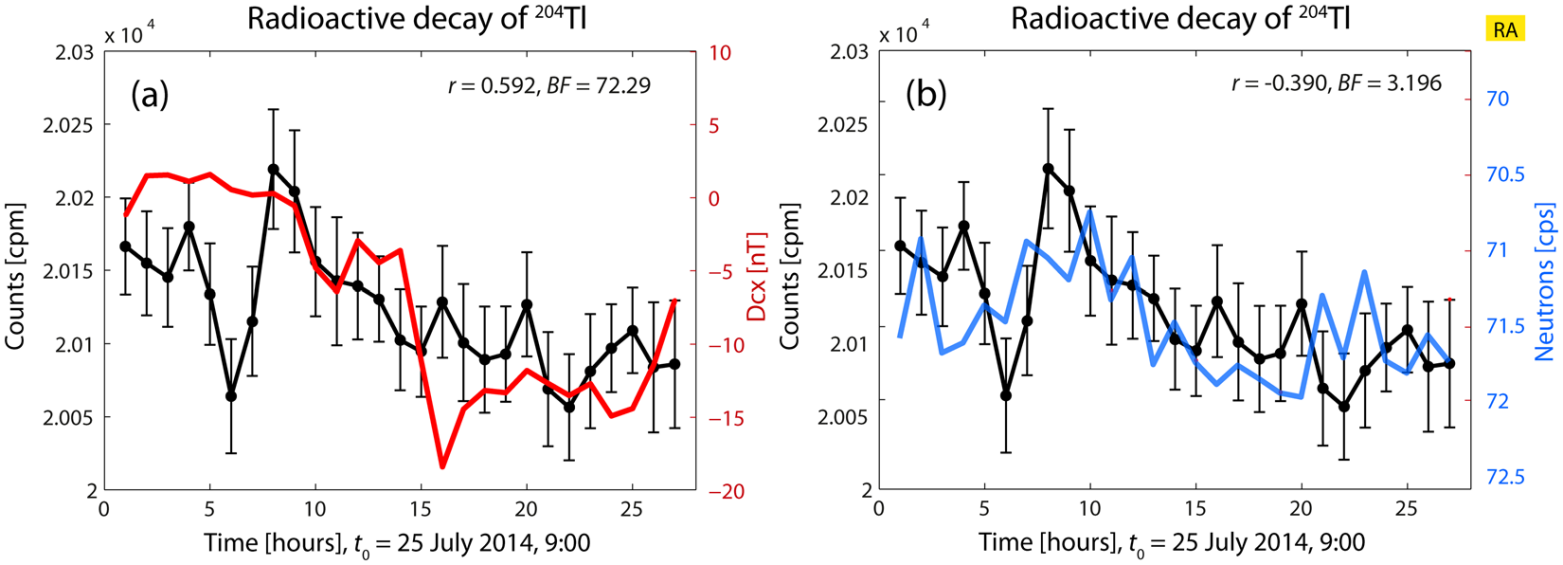
Decay rate of ^204^Tl vs. Dcx (**a**) and neutron counts (**b**). Counts in this figure increase about 1.1% relatively to the values outside the box. RA: reversed axis scaling.

Figure 5 shows the decay behavior of a preparation of ^204^Tl. These decay data were presented in Fig. 5 of Ref. ^1^. Counts increased when placed inside the box by about 1.1% relatively to the first data obtained outside the enclosure (20000 cpm).

The decay data are statistically significantly correlated with GMA (decay vs. Dcx: *r* = −0.592, BF = 72.29) and CRA (decay vs. neutron counts: *r* = −0.390, BF = 3.196). It should be noticed that here again (a case where both parameters are correlated to cpm), one of them is anti-correlated, as in Fig. 2.

Regarding subfigure (a), decay data oscillate and yield statistically different values; Dcx osculates within the range [2, −18] nT.

Regarding subfigure (b), the neutron counts oscillate in a range lower than 2 cps and similarly as in Fig. 3. As in this figure, the correlation between decay and neutron counts is weak. One could say that all this facilitates Dcx to be the leading factor.

To sum up, in this experiment, the decay rate is increased, Dcx is oscillating broadly, and some neutron counts are close to 72 cpm but not so many as in Fig. 1. These circumstances seem to favor that decay rates are correlated with both Dcx and neutron counts, but keeping in mind that cpm and N are anti-correlated; this is a similar result as in Fig. 2, and seems to show that when both parameters Dcx and N are correlated to cpm, one of them is anti-correlated.

#### Radioactive decay of ^90^Sr/^90^Y *vs*. geomagnetic and cosmic-ray activity

**Figure 6:**
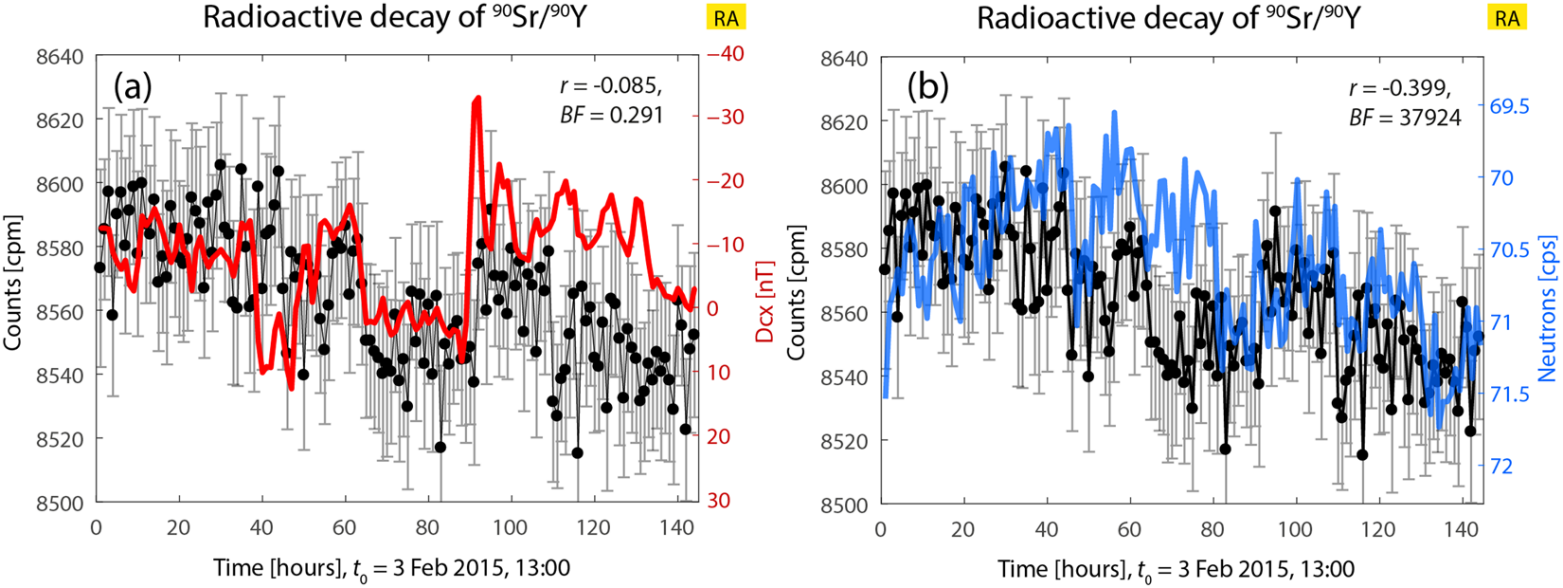
Decay rate of ^90^Sr/^90^Y vs. Dcx and neutron counts; (**a**) uncertain good correlation between both variables due to the high transient, (**b**) Good correlation exists in (**b**). In (**a**) and (**b**): decay rates oscillations are >4 cpm. In (**a**) and (**b**): decay data oscillations are >7 cpm.

Figure 6 shows the correlations found between decay rates of the ^90^Sr/^90^Y source with GMA and CRA. In this experiment, no statistically significant correlation with GMA but with CRA was found (decay vs. Dcx: *r* = −0.085, BF = 0.291; decay vs. neutron counts: *r* = 0.399, BF = 37924). Visually, a correlation between GMA and decay in Fig. 6(a) can be clearly seen, but the correlation analysis is not able to capture this correlation correctly. The decay data in Fig. 6(a,b) are shown in Fig. 6(c) in^1^.

In Fig. 6, the decay rate oscillates with a magnitude of more than 6 σ_m_; they are also increased and show a slight downward trend. The Dcx index oscillate between positive and negative values (approx. range [+10, −35] nT) and the neutron counts fluctuate with decay counts in the range [69.5, 71.5]. Under these circumstances, there is not a good correlation with Dcx, but with the neutron counts (anti-correlations). However, the lack of correlation of decay rate with Dcx is most probably due to the strong transients.

Regarding the up till now observed downward or upward trend, in this case there is a slight downward trend (in decay data), whereas an upward trend was seen in Fig. 2. From these comparisons, it could be hypothesized that the first decisive factor is not only an upward or downward trend, but that the decay rates are: increased (or increasing), or not increased but significantly oscillating, and not highly reduced as in some previous (and in the next) case.

The results regarding the Bayes factor shown in this figure can be associated to the following: Fig. 6(a) shows a very high transient, which prevents a correct calculation. The result referred to Fig. 6(b) coincides with preparation increased cpm, and strong fluctuations of the neutron counts.

#### Remarks about the correlations between measured radioactive decay and geomagnetic as well as cosmic ray activity

The correlation analysis based on the experimental data shown in the previous seven figures yielded that only in three (or four) out of eight cases there was a statistically significant correlation between decay data and GMA. Figures 2(a), 3(a) and 5(a) show good correlations (Fig. 6(a) might be regarded as a positive result, but it should be tested with another tools). From Figs. 2 and 5 it seems that when both GMA and CRA are correlated at the same time, with decay rates, one of them is correlated and the other variable is anti-correlated.

At first sight, the presence or absence of a correlation seems not to be related to the particular radioactive material, since some cases with ^226^Ra show a correlation but not always. Rather, from the limited data available, it seems more likely to be dependent on a combination of factors, namely, the increased or decreased state of decay rates, and on the type of variability, as well as the absolute state, of the GMA and CRA. From the small amount of available data, it can be hypothesized that a favorable situation for the presence of a correlation of decay rates and GMA/CRA occurs when the measurement inside the MFC yields more counts than outside.

Regarding the CRA indexed by the neutron counts, in five out of seven cases there was a statistically significant correlation. Figure 2(b) shows a very good correlation, and good correlations appear for Figs. 1(b), 5(b), 6(b). Therefore, making use of the available data, the probability of observing a correlation is higher between CRA and decay rates.

Moreover, as in the case of Dcx, correlations between decay rates and neutron counts seem to be also dependent on the state of increased or decreased state of decay rates, and on whether it is correlated with Dcx or not: when Dcx and neutron rates are not correlated (and cpm are increased), one of the two factors (the most dominant) *can be* correlated with decay rates. The neutron counts seem to be correlated with cpm if Dcx does not oscillate sufficiently (broad oscillations surpassing the limits of the low values of Dcx), and when the neutron counts are close or higher than 72 cpm. Since the quantification of these aspects of the oscillations is needed, an analysis with other tools will be reported in a next paper.

**Figure 7:**
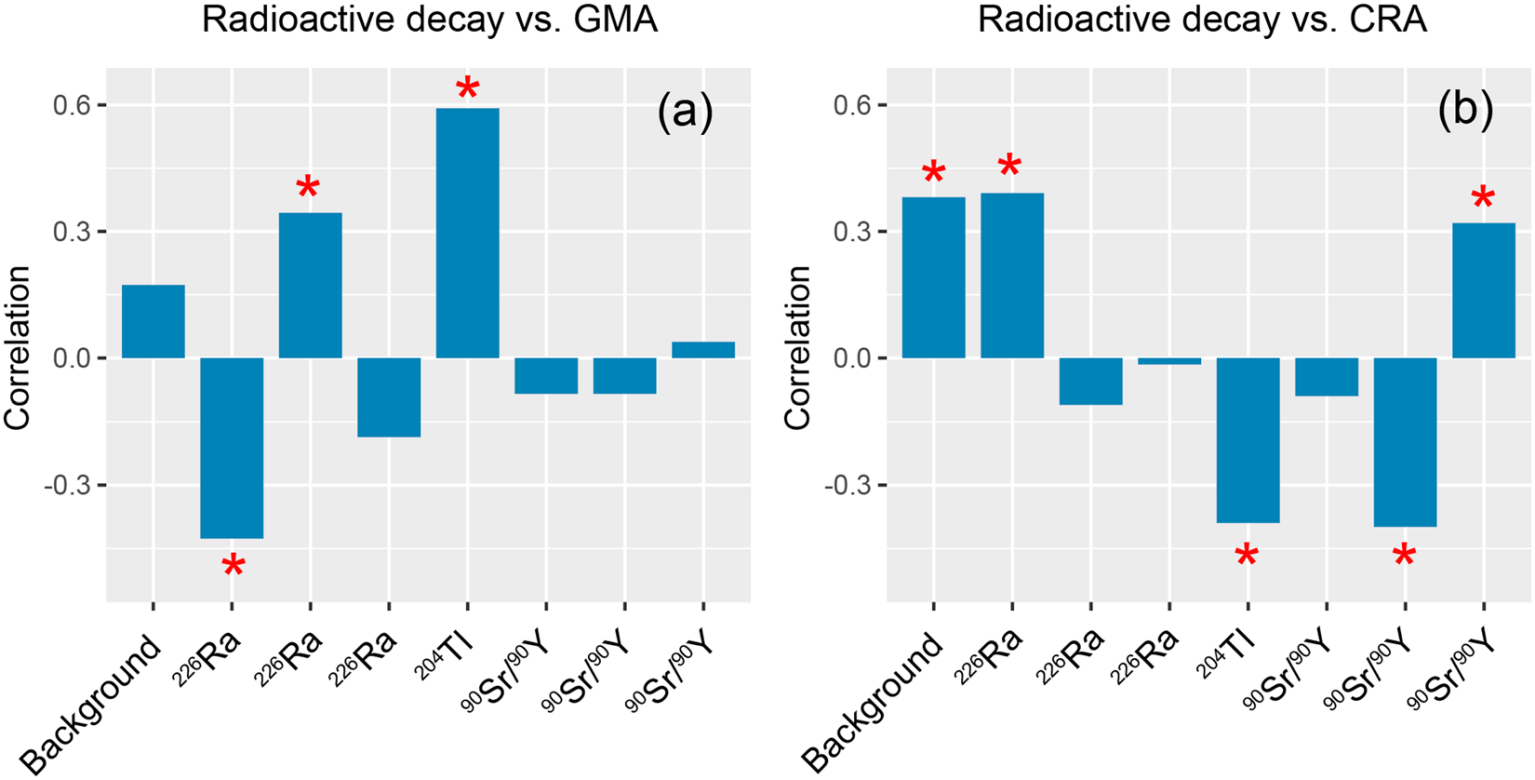
Summary of the correlation strength between radioactive decay and GMA (**a**) as well as CRA (**b**) based on the analysis presented in sections 3.1.1–3.1.4. *Statistically significant (BF > 3).

Figure 7 gives an overview of the correlation strengths of radioactivity with GMA and CRA for the experiments discussed so far.

We can conclude that in some experiments performed there is a clear correlation of the decay activity with space weather, i.e. GMA and/or CRA. However, it must be also pointed out that the correlations are sometimes positive and sometimes negative. All this makes the presented results difficult to understand since there are various circumstances under which those correlations take place or not.

Yet, not only the question of why decay rates can be so strongly increased or reduced *inside* the MFC remains unanswered and the ultimate reason unknown, but now additional questions arise, specifically, why sometimes correlations exist and sometimes not, and the question about the physical mechanism behind these observations. In this respect, as already mentioned in our previous paper^42^, it seems apparent that there exists of a link between the primary physical magnitudes (Dcx and neutron counts) and the responses of the sensors (Geiger-Müller counter and the capacitor), when the above described circumstances converge. The interested reader can find a particular interpretation of the results in the work of W. Reich^56^.

It can be argued that sometimes the correlations are not be observed, but this is not necessarily a lack of consistency in the observed coincident circumstances: it seems only to mean a lack of conditions for such correlations to occur.

It was also shown in the initial report by us^1^ that capacitance variations were a factor that explained part of the decay measurements variability, but at the same time, this factor alone could not explain all the observed variability. Besides, the reason for this change is unknown. Similar as it was done by Fischbach *et al*.^57^, it is only possible to make conjectures about the reasons which in turn match with the observed experimental results. Moreover, capacitance measurements can also be correlated with GMA and CRA, as shown in the next section.

### Capacitance measurements vs. geomagnetic and cosmic-ray activity

As follows, figures about the found (and not found) correlations between capacitance variations and both Dcx and neutron counts are shown and discussed. For the following discussion, the results in previous sections will be useful because capacitance and decay rates anomalous variations are related to each other.

#### Capacitance at 10 kHz

**Figure 8:**
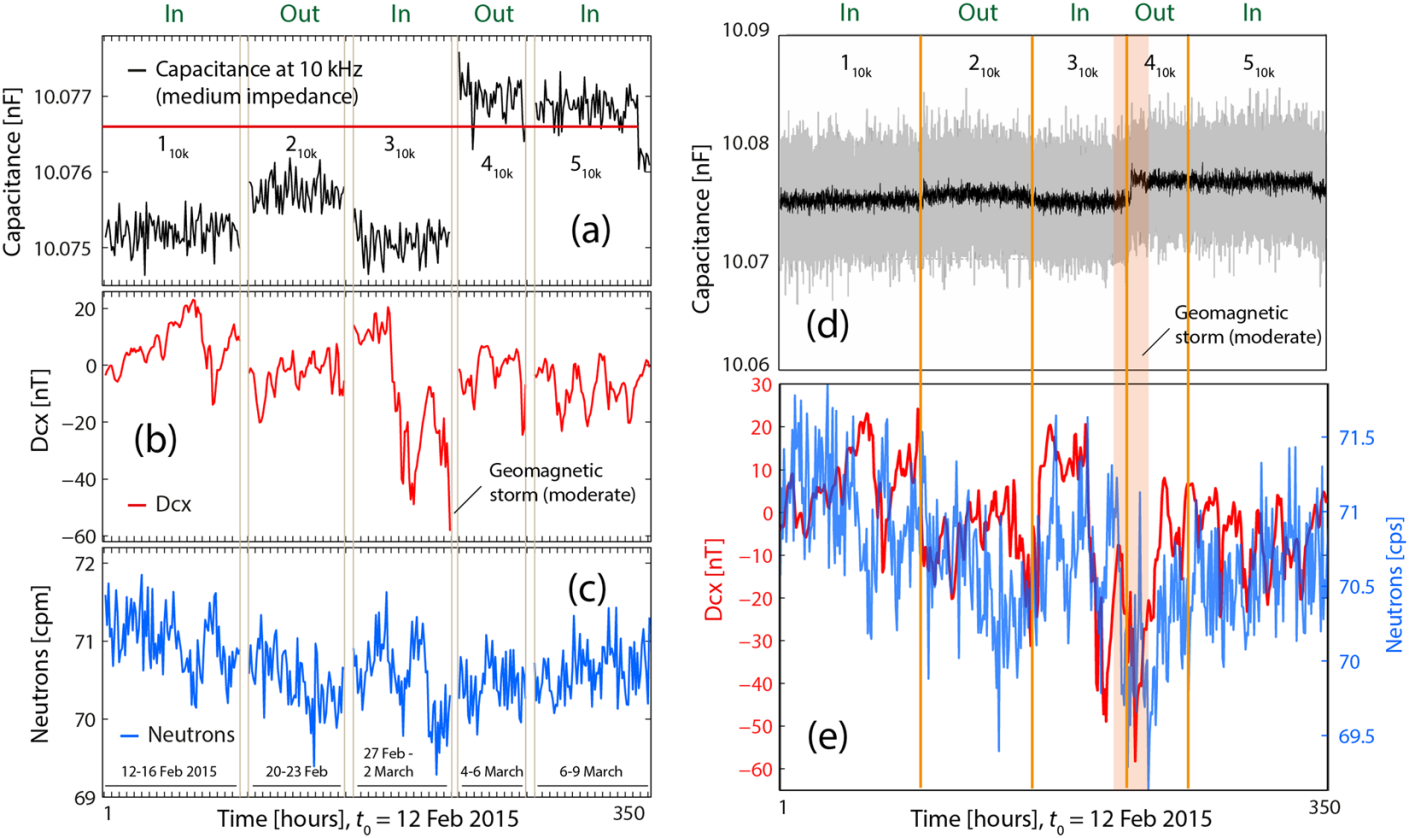
Capacitance data at 10 kHz *vs* Dcx and neutron counts. Capacitance data (**a**) vs Dcx index (**b**) and vs. neutron counts (**c**); there is no correlation between any pair of data sets. Besides, capacitances are close to the value outside the box, but below or above this value. (**d,e**) Capacitance data vs Dcx and neutron counts. The stronger jump coincides with a moderate geomagnetic storm.

**Figure 9:**
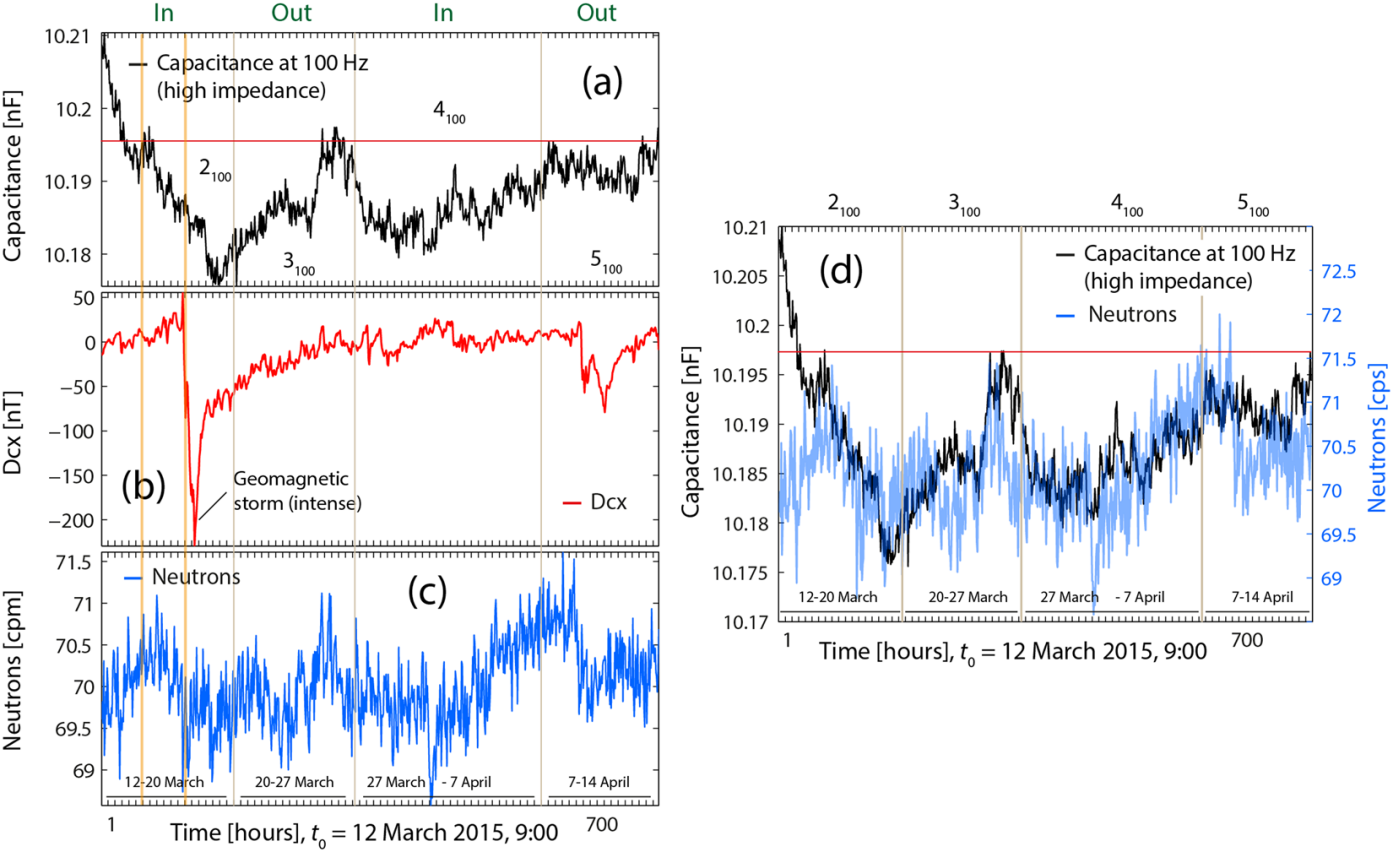
Capacitance data at 100 Hz vs Dcx and *vs* neutron counts. Capacitance (**a**) *vs* Dcx (**b**) and *vs* neutron counts (**c**). The intense geomagnetic storm, see (**b**), precedes the last capacitance drop in the first descending ramp, see (**a**). (**d**) Capacitance is not correlated with neutron counts (and not with with Dcx) during about the first part of the first descending ramp.

The capacitance measurements at 10 kHz presented in Fig. 8(a) (see also Fig. 9(a,b) in^1^) are not correlated with the overall fluctuations of Dcx and neutrons counts.

First of all one can observe that in this figure, the capacitance values of the first three means (intervals 1_10k_, 2_10k_ and 3_10k_) are below and close (i.e. at 0.0159%) to the *initial* value outside the box (which is 10.0766 nF, as shown by the red line in Fig. 8 and in^1^), whereas the two last means (intervals 4_10k_, 5_10k_) are much closer (0.004%), but above the red line.

Capacitance values during periods 1_10k_ and 3_10k_ (inside the box), which are slightly decreased relatively to the initial value, can be associated with higher than normal decay rates (which presumably would have been obtained if measurements had been performed during those periods). Dcx values are relatively close to and oscillating around 0 nT during intervals 1_10k_, and 3_10k_ without strong positive and negative variations; therefore, and as seen in Figs. 1 and 6, all this supposedly implies lack of correlation between decay and Dcx.

Besides, during the intervals 4_10k_ and 5_10k_, capacitance is higher than normal, which is equivalent to a reduction of the recorded decay rates: according to what has been seen previously, no correlation between cpm and Dcx (or neutron counts) would exist. The current conditions should be enough for the lack of correlations between capacitance and both Dcx and N.

Neutron counts variations are also not correlated with Dcx in none of the intervals where the frequency was 10 kHz. During periods 1_10k_, and 3_10k_ the capacitance decrease was very low, and the situation was similar to that of Fig. 3(b), and during interval 5_10k_, the equivalency to a reduced counts situation also corresponds to a lack of correlation.

It is also noticeable that the capacitance values outside the box (see intervals 2_10k_ and 4_10k_), remain in quite stationary states below and above the state it would hold outside the box.

The low jump after the first extraction (from 1_10k_ to 2_10k_) cannot be correlated with neither Dcx nor with neutron count variations. Rather, it can be associated with a time-lag effect similar to the one represented in Figure 10 in^1^ (ascending ramp in the “OUT” section), i.e. it represents a slow tendency to return to outside values after the extraction from the box.

It can also be seen in Fig. 8 that the observed jump to a higher regime (period 4_10k_ outside), and in the way it happened, took place after the moderate geomagnetic storm, which occurred during the 3_10k_ measurements. This jump coincides with the time of the (second) extraction of the source from the box (as in Fig. 4(a) of^1^) and again the change to a higher regime was not immediate. We can assume that decay rates which could have been measured during the transition from 3_10k_ to 4_10k_ would have experienced a significant drop in cpm mesurements. Besides, it reminds the transient from 10.14 nF to 10.21 nF observed in Fig. 9.

It is also anomalous that capacitors values outside the box (periods 2_10k_ and 4_10k_) are not the same as in the first measurements at the outside (Fig. 8 in^1^).

Capacitance values during period 5_10k_ are not correlated with none of the two indices. This could be interpreted in the same way as before: cpm would have been very close to initial values (but decreased), Dcx values slightly oscillating and close to 0 nT and neutron count fluctuations are not correlated with Dcx; besides, and since decay rates are not increased (but slightly decreased) and neutron count oscillations are small, no correlation between cpm and neutron counts would have been observed.

#### Capacitance at 100 Hz

The analysis of the capacitance data at 100 Hz (Fig. 9) shows that there was a strong geomagnetic storm during the capacitance measurements inside the box (see Fig. 9(b)). In this figure, one can observe a drop of capacitance very shortly after the onset of the storm (Fig. 9(a)), and one can ask whether such storm was the precursor signal of this last capacitance drop. Conversely, is the (previous) first part of the descending ramp in (a) (from 10.21 nF to10.185 nF), the precursor signal of the *strong* geomagnetic storm? Some works refer to such kind of effects^49^.

Regarding the lack of correlation between capacitances values and Dcx (in Fig. 9(a,b)) two considerations can be made. The capacitance value of the reference (at 100 Hz and prior to any interaction with the box), was 10.1966 nF (see Fig. 8 in^1^ and red line in Fig. 9a). On the one hand, since the capacitances in the first interval inside the box (2_100_, part of the descending ramp from 10.21 nF until 10.1966 nF) are above this value, they could be the expression of decay rates lower than the values outside the box. On the other hand, most of Dcx values were quite stationary (range [0, 20 nT]) and relatively close to 0 nT. In previous sections it could be seen that under these two circumstances there was no correlation with Dcx.

The second appreciation is that the second part of interval 2_100_ (i.e., C < 10.1966 nF, and the rest of the capacitance values (in Fig. 9) are close or below the reference value (the latter equivalent to increased cpm –increased pulses height), but at the same time, Dcx values are quite close to 0 nT (except those during the storm), which was also a circumstance under which there was no correlation (see Fig. 1).

With regard to the existing correlation between the capacitance variations and neutron counts, it can be seen in Fig. 9(b) that the correlation is strong after the second part of period 2_100_. This coincides with capacitance values close or lower than the value of reference (which in turn can be mostly related to “normal” or increased decay rates values); this is also similar to what seen in Fig. 1.

#### Remarks about the capacitance measurements and geomagnetic as well as cosmic ray activity

Based on the cases discussed above we can conclude that the appearance of correlations of capacitance variations with GMA/CRA changes is similar to the appearance of correlations of radioactive changes with GMA/CRA variations. This is so because decay rates variations are related to capacitance variations, as shown in^1^. It must be clarified which physical reasons is responsible for those coincidences. As in the case of decay rates, the aforementioned coincidences are not an explanation of the observed effects.

Although an explanation of the observed effects (based on first principles) has not been given, some hint or clue appears based on the capacitance variation. Since this apparent variation refers to changes in dielectric polarizability, it is necessary to further analyze this aspect. An explanation of what happens in the observed anomalous decay rates measurements could eventually be obtained from this clue, if the same factor, which changes the polarizability, would be affecting other constituents of the detection system. This will be analyzed in a later study.

## General remarks and conclusions

The results of anomalous radioactivity and/or capacitance measurements inside an MFC are sometimes correlated with GMA and CRA, i.e. space weather. From the available data, we observed that those correlations exist (or do not exist) under certain circumstances. These circumstances can be summarized as follows:

(i) In the cases studied, no correlations with space weather were evident when the decay rates (or capacitance measurement) were the same as measured outside of the MFC, or if they are significantly decreased (increased) with regard to those values outside the enclosure. In such case, it is irrelevant how Dcx and the neutron counts behave.
(ii) A general condition for the occurrence of a correlation between decay rates (or capacitance measurements) and space weather is that the decay measurements (capacitance measurements) taken inside the box must be greater (lower) inside than those taken outside.
(iii) In case of no difference of the decay data with respect to the measurement inside or outside, the inside measured data must show a clear fluctuation/oscillation so that a correlation with space weather variables is evident (can become apparent).
(iv) The analysis showed that GMA and CRA are sometimes correlated. In case of a correlation, the decay values are likely to be correlated with decay data if conditions (ii) or (iii) were satisfied. When GMA and CRA are not correlated, the decay data can be correlated with either GMA or CRA. Thus, a correlation between decay data and GMA prevails when conditions (ii) and (iii) hold and when Dcx oscillate significantly around 0 nT, with noticeable negative and positive values. A correlation between decay data and CRA prevails when conditions (ii) and (iii) hold, and when the neutron counts oscillates more intensively than Dcx (or when it takes values close and/or higher than 72 cps).
(v) As a consequence of (i), it can be observed, that correlations between capacitance values (at 100 Hz) and neutron counts are not observable when capacitance values are increased relatively to their initial values (see Fig. 9d, first half part of the first descending ramp). However, correlation with neutron counts can exist when capacitance values are reduced with respect to their initial values.

The results raise the question whether capacitance changes (at 100 kHz and/or at 100 Hz) might be a response to strong geomagnetic storms (see interval 2_100_ in Fig. 8). In other words, whether the magnetic storm could be the precursor signal of some observed capacitance changes, and therefore of decay rates variations.

The finding described in this paper reveals that there exists a link between space weather (i.e. GMA and CRA) and the sensors’ responses inside the (and thanks to) the MFC. It is an open question why this interaction exists and what the underlying physical mechanism is. Additional investigations are needed to measure additional physical parameters related to the measurement setup as well as factors from the environment.

In our study we used the isotopes ^226^Ra, ^204^Tl, ^90^Sr/^90^Y and ^137^Cs. Since previous studies found that the probability of observing non-random fluctuations in radioactive decay data also depends on the specific isotope (e.g.^25,35,58,59^), it would be important to investigate a great variety of radioactive isotopes with the approach and method we used in our study. This should be done in order to systematically investigate which isotope’s decay variability is correlated with GMA and CRA.
